# Emergency vs elective ureteroscopy for a single ureteric stone

**DOI:** 10.1080/2090598X.2020.1813004

**Published:** 2020-08-25

**Authors:** Abdullatif Al-Terki, Majd Alkabbani, Talal A. Alenezi, Tariq F. Al-Shaiji, Shabir Al-Mousawi, Ahmed R. El-Nahas

**Affiliations:** a Urology Unit, Al-Amiri Hospital, Kuwait City, Kuwait; b Urology and Nephrology Center, Mansoura University, Mansoura, Egypt

**Keywords:** Emergency, ureteroscopy, renal colic, ureter, ureteric stone, calculi

## Abstract

**Objective:**

To compare emergency with elective ureteroscopy (URS) for the treatment of a single ureteric stone.

**Patients and methods:**

The files of adult patients with a single ureteric stone were retrospectively reviewed. Patients with fever or turbid urine on passage of the guidewire beside the stone underwent ureteric stenting or nephrostomy drainage. Patients who underwent URS were included and divided into two groups: the emergency (EM) Group, those who presented with persistent renal colic and underwent emergency URS within 24 h; and the elective (EL) Group, who underwent elective URS after ≥14 days of diagnosis. Patients with ureteric stents were excluded. The technique for URS was the same in both groups. Safety was defined as absence of complications. Efficacy was defined as the stone-free rate after a single URS session.

**Results:**

From March 2015 to September 2018, 179 patients (107 in the EM Group and 72 in the EL Group) were included. There were significantly more hydronephrosis and smaller stones in the EM Group (*P* = 0.002 and *P* = 0.001, respectively). Laser disintegration was needed in more patients in the EL Group (83% vs 68%, *P* = 0.023). Post-URS ureteric stents were inserted in more patients in the EM Group (91% vs 72%, *P* = 0.001). Complications were comparable for both groups (4.2% for EL and 5.6% for EM, *P* = 0.665). Stone-free rates were also comparable (93% in the EL Group and 96% in the EM Group, *P* = 0.336).

**Conclusions:**

Emergency URS can be as safe and effective as elective URS for the treatment of a single ureteric stone if it is performed in patients without fever or turbid urine.

**Abbreviations**: EL Group: elective group; EM Group: emergency group; KUB: plain abdominal radiograph of the kidneys, ureters and bladder; MET: medical expulsive therapy; NCCT: non-contrast CT; SFR: stone-free rate; SWL: shockwave lithotripsy; URS: ureteroscopy

## Introduction

The most common presentation of ureteric calculi is acute renal colic. This severe pain episode urges the patient to seek medical advice immediately [1] and it is a leading urological cause of emergency department visits [2]. Management of acute renal colic secondary to ureteric calculi starts with analgesics to control pain [3]. If analgesics fail to control pain or there are complications of obstruction, such as fever or acute kidney injury, upper tract drainage with a nephrostomy tube or ureteric stent is required [4]. Elective treatment modalities for ureteric calculi include medical expulsive therapy (MET), extracorporeal shockwave lithotripsy (SWL), and ureteroscopy (URS). The choice of a certain treatment depends on the patient’s presentation, comorbidities, renal and stone characteristics, availability of instruments, and surgeon experience [5].

Emergency active treatments of ureteric calculi in the form of SWL [6,7] or URS [8–10] within 48 h after a colic episode have been reported. The advantage of emergency active treatment is based on immediate retrieval of the obstructing stone, therefore, decreasing the number of surgical interventions (i.e. relief of obstruction in one session and then treatment of the stones in the second). This will result in reduction of follow-up visits, radiation exposure and ultimately the costs [9]. Another value of emergency active treatment is decreasing morbidity and mortality of delayed treatment [4]. However, emergency treatment is suitable only for a certain group of patients who have no complications at presentation or who are not deemed at high risk of development of complications [11].

Emergency URS has gained in popularity in recent years because of the advances in intracorporeal laser lithotripsy and development of tipless nitinol baskets, which have led to the increased safety and efficacy of URS in the treatment of ureteric stones. There is still debate about the role of emergency URS in patients who present with acute calcular obstruction, despite comparable stone-free rates for emergency and elective URS [12], and international guidelines are not specific on this issue. The main fear of some is derived from the theoretical increased risk of complications such as ureteric injury or postoperative fever.

The present study was conducted to compare the results of emergency and elective ureteroscopy (URS) for the treatment of a single ureteric stone.

## Patients and methods

### Patients

The files of adult patients with a single ureteric stone were retrospectively reviewed. In our hospital, the protocol for management of patients with complications of obstruction (e.g. fever or sepsis) due to ureteric stones implies drainage of the upper urinary tract with either a ureteric stent or nephrostomy tube. Moreover, when turbid urine is drained during the passage of the guidewire beside the ureteric stone, a ureteric stent was inserted and URS was not attempted. The study included consecutive adult patients who underwent URS for the treatment of a single ureteric stone diagnosed by non-contrast CT (NCCT). Patients with preoperatively placed ureteric stents were excluded.

The study compared two groups: the emergency (EM) Group, which included patients who underwent emergency URS within 24 h of presentation to the emergency department with persistent renal colic; and the elective (EL) Group, which included patients who underwent elective URS who were admitted through the outpatient appointment system after ≥14 days from diagnosis. The period of 14 days was chosen because it is the initial period for evaluation of MET in our hospital. If there was no downward progression of the stone, the patient underwent elective URS. Preoperative assessment included urine analysis, full blood count, serum creatinine, and NCCT.

### Operative details

All patients received intravenous ceftriaxone with induction of anaesthesia. Retrograde pyelography was not routinely done. The technique of URS was the same for both groups. Under general anaesthesia, a long semi-rigid ureteroscope (Richard Wolf, Knittingen, Germany) was used. A flexible ureteroscope (FlexX2, Karl Storz Endoskope, Tuttlingen, Germany) was available in the operating room in case the rigid ureteroscope could not reach the upper ureteric stone or stone fragments migrated to the kidney during laser disintegration. No anti-retropulsion devices were used. A small ureteric stone was extracted using a tipless nitinol basket (Dormia No-Tip 2.2 F, Coloplast, Humlebaek, Denmark). Large ureteric stones were fragmented with holmium laser (Calculase®; Karl Storz Endoskope) then fragments were retrieved with the basket. Lithotripsy was used if the surgeon judged that the stone could not be extracted safely as one piece. Laser power was adjusted to 0.8–1 J and 6–10 Hz. At the end of the procedure, an externally draining ureteric catheter was placed for 24–48 h, unless there was stone impaction or ureteric injury where a ureteric stent was placed for 2–4 weeks.

### Postoperative evaluation

A plain abdominal radiograph of the kidneys, ureters and bladder (KUB) was taken on the first postoperative day to confirm the correct positioning of the ureteric catheter or stent. Complications were recorded and classified based on the modified Clavien system. The stone-free rate (SFR) was evaluated with KUB for radiopaque stones and NCCT for lucent stones 2–4 weeks after URS. Safety was defined as absence of complications, while efficacy was defined as SFR after a single URS session.

### Statistical analysis

Data were stored and analysed with the Statistical Package for the Social Sciences (SPSS®), version 20 (SPSS Inc., IBM Corp., Armonk, NY, USA). Both groups were compared for preoperative characteristics (age, gender, stone side, size, level, hydronephrosis, and creatinine), operative details (methods of stone disintegration and retrieval), postoperative hospital stay, and outcomes (SFR and complications). The chi-square test was used to compare nominal data, while the independent sample *t*-test or Mann–Whitney *U*-test was used to compare continuous data. A *P < *0.05 was considered statistically significant.

## Results

From March 2015 to September 2018, 741 patients underwent URS for treatment of ureteric stones. After exclusion of elective cases who had pre-URS ureteric stents, the study included 179 patients (107 in the EM Group and 72 in the EL Group). The preoperative characters are summarised in Table 1. There was significantly more hydronephrosis, higher creatinine levels and smaller stones in the EM Group.

**Table 1.** Preoperative characteristics of the study groups

Characteristic	EL Group (*N* = 72)	EM Group (*N* = 107)	*P*
*N* (%):			
Gender Male Female	49 (68) 23 (32)	88 (82.2) 19 (17.8)	0.028*
Stone history First time Recurrent	62 (86) 10 (14)	94 (88) 13 (12)	0.733*
Hydronephrosis No or mild Moderate	65 (90.3) 7 (9.7)	76 (71) 31 (29)	0.002*
Stone side Right Left	45 (62.5) 27 (37.5)	55 (51.4) 52 (48.6)	0.143*
Stone level Proximal ureter Distal ureter	26 (36) 46 (64)	25 (23.4) 82 (76.6)	0.064*
Mean (SD):			
Age, years	40.8 (11.7)	43.1 (12.8)	0.226^&^
Creatinine level µmol/L mg/dL	79.9 (20.6) 0.91 (0.23)	133.1 (57.4) 1.5 (0.65)	<0.001^&^
Stone length,mm	9.1 (3.7)	7.1 (2.9)	<0.001^&^

*Chi-square test; **^&^**Independent-samples *t*-test

Intra- and postoperative data are summarised in Table 2. Laser disintegration was needed in significantly more patients in the EL Group (*P* = 0.023) and basket retrieval of the stones was required significantly more in the EM Group (*P* = 0.037). Post-URS JJ stents were inserted for more patients in the EM Group (*P* = 0.001).

**Table 2.** Intraoperative details and postoperative outcomes of the study groups

Characteristic	EL Group (*N*= 72)	EM Group (*N*= 107)	*P*
*N*(%):			
Laser lithotripsy	60 (83.3)	73 (68.2)	0.023
Basket retrieval	44 (61)	81 (75.5)	0.037
Post-URS renal drainage Ureteric catheter JJ stent	20 (27.8) 52 (72.2)	9 (8.4) 98 (91.6)	0.001
Complications: Intraoperative Postoperative: Clavien Grade II Clavien Grade III	3 (4.2) 0 3 1 2	6 (5.6) 1 5 4 1	0.665
Stone free	67 (93.1)	103 (96.3)	0.336
Hospital stay, days, median (range)	1 (1–10)	1 (1–6)	0.866^#^

^#:^Mann–Whitney *U*-test.

Complications were comparable between the groups (4.2% for EL and 5.6% for EM, *P* = 0.665; Table 2). Ureteric perforation during laser lithotripsy in one patient in the EM Group was treated with JJ stenting. Febrile UTI in one patient in the EL Group and three in the EM Group were treated with antibiotics. Upper tract obstruction after removal of the ureteric catheter in one patient in the EL Group needed JJ stenting. Haematuria in one patient in the EM Group was treated with a blood transfusion. Ureteric stricture at the site of stones was observed in one patient of in each group after 2 months; they were successfully treated with laser endoureterotomy.

As shown in Table 2, the SFRs were comparable between the groups (93% in the EL Group and 96% in the EM Group, *P* = 0.336). The nine patients with residual stones were treated with re-URS in five, SWL in three and mini-percutaneous nephrolithotomy in one.

## Discussion

The reluctance to perform emergency active intervention in patients with acute colic due to ureteric stones appears to be based on the fear of the development of complications. The results of the present study showed that emergency URS can be safely performed in a select group of patients, as the complication rates were comparable to elective cases (5.6% vs 4.2%, *P* = 0.665). The 5.6% complication rate in the present study is comparable to 5% complication rate reported by Zargar-Shoshtari *et al*. [10], who evaluated 394 cases. The complication rate in the present study is slightly lower than 7.6% reported by Picozzi *et al*. [12] in a meta-analysis of 681 emergency URS cases, and the 8.7% reported by Arcaniolo *et al*. [13] in their cumulative analysis of comparative studies that included 526 cases of emergency URS. This difference can be explained by the careful selection of emergency URS patients in the present study. We did not perform emergency URS for cases that showed infected urine on passage of the guidewire beside the stone and cases with multiple stones, as they may need more operative time, which increases the risk of complications. Selection of cases suitable for safe emergency URS (uncomplicated presentation and no risks of complications) is the cornerstone in decreasing complications of emergency URS [11]. Recently, there was a report of performing emergency URS in patients who presented with urosepsis [14]. This pilot study was criticised with a small sample size of 13 patients in each group, and therefore, their conclusions cannot be generalised.

The severities of complications in the present study were also comparable for both groups (Table 2) and no patients developed complications of Grade 4 or 5 using the modified Clavien classification. Such findings have also been reported by previous studies [9–11,15,16]. One of the causes of decreasing severity of complications in URS, in addition to patients’ selection, is the use of laser lithotripsy for large stones. Therefore, ureteric avulsion is avoided.

The SFRs of emergency and elective URS in the present study were comparable (96% and 93% respectively, *P* = 0.336). This was also reported in all previously published comparative studies [9,11,13,15,16]. The reported SFR of emergency URS ranged between 81% and 93% [8,9,11–13,15–18]. We achieved a better SFR (96%) due to the use of laser lithotripsy in all cases. The most obvious advantage of laser over pneumatic lithotripsy (that was used in some previous studies) is the decreased rate of stone retropulsion. The higher SFR can also be attributed to the high case volume of URS per year in our hospital (~164 cases/year). Kandasami *et al*. [19] reported in the global ureteroscopy study conducted by the Clinical Research Office of the Endourological Society (CROES) that the probability of a stone-free outcome is significantly increased in hospitals with a high case volume per year. Another cause was the small size of stones in the present study, as it has been reported that the stone diameter affects SFR of emergency URS. Picozzi *et al*. [12] in their meta-analysis concluded that an increase of stone size by 1 mm resulted in a reduction in the SFR by 5–8%. Similarly, Zargar-Shoshtari *et al*. [10] found that the mean stone size in successful emergency URS was 7 mm compared to 9 mm in those with an unsuccessful outcome.

The retrospective design is the main limitation of the present study, as some data were not available such as operative time. We tried to do a fair comparison by excluding elective cases with an already present ureteric stent because all emergency cases had no stents. However, there was still some inevitable selection bias, as the stones were significantly smaller in the EM Group. This led to more use of baskets in the EM Group and more use of laser lithotripsy the EL Group. Also, the EM Group had more hydronephrosis than the EL Group, which resulted in the insertion of more ureteric stents (92% vs 72%). Another significant difference was observed in serum creatinine levels, as it was significantly higher in the EM Group (1.5 vs 0.9 mg/dL). This is expected, as acute renal colic can be associated with nausea and vomiting that may cause dehydration. We performed emergency URS in these patients because Abdel-Kader [20] reported the safety of emergency URS in patients with calcular anuria and high serum creatinine at a mean level of 3.5 mg/dL.

## Conclusions

Emergency URS can be as safe and effective as elective URS for the treatment of a single ureteric stone if it is performed in patients without fever or turbid urine.
